# Expression profile of tsRNAs in white adipose tissue of vitamin D deficiency young male mice with or without obesity

**DOI:** 10.1038/s41598-024-77910-9

**Published:** 2024-11-11

**Authors:** Qiaowei Jia, Yan Zhao

**Affiliations:** 1 Department of Clinical Nutrition, Jiangsu Province Hospital and the First Affiliated Hospital of Nanjing Medical University, Nanjing, 210029 Jiangsu Province China; 2 Department of Cardiovascular Medicine, Jiangsu Province Hospital and the First Affiliated Hospital of Nanjing Medical University, Nanjing, 210029 Jiangsu Province China

**Keywords:** TsRNA expression profile, TiRNAs, TRFs, Obesity, Vitamin D, Adipose tissue, RNA, Gene expression analysis, High-throughput screening

## Abstract

The expression of tsRNA in white adipose tissue (WAT) of VD deficiency male mice with obesity has not been reported. The healthy male C57BL/6J mice aged 4-6 weeks were divided into 4 groups according to the VD_3_ and fat energy supplement in daily diets. The qPCR verification further demonstrated that tRF5-20-HisGTG-3 were significantly up-regulated and mt-tRF3a-ProTGG was significantly down-regulated not only in HFVDD vs HFVDS, but aslo in HFVDD vs ConVDS. tRF5-22-CysGCA-27 were significantly up-regulated and mt-5'tiRNA-32-SerTGA, mt-5'tiRNA-33-SerTGA and mt-5'tiRNA-33-AlaTGC was significantly down-regulated only in HFVDD vs ConVDS. Enrichment analysis of the qPCR verified DE tsRNAs showed that the 3 up-regulated tsRNAs seemed to be associated with FoxO signaling pathway, GnRH secretion, 2−Oxocarboxylic acid metabolism, Autophagy-animal, Glucagon and insulin signaling pathway, while 4 down-regulated tsRNA seemed to be associated with cell communication, primary metabolic process, metabolic process, response to stimulus, multicellular organismal process, cellular metabolic process, cellular process and biological regulation. The tsRNAs were differentially expressed in VD deficiency with obesity, especially tRF5-20-HisGTG-3, tRF5-22-CysGCA-27, tRF3a-GlyGCC-1, mt-5'tiRNA-33-AlaTGC, mt-5'tiRNA-33-SerTGA, mt-5'tiRNA-32-SerTGA and mt-tRF3a-ProTGG. These tsRNAs seemed to be associated with FoxO signaling pathway, GnRH secretion, 2−oxocarboxylic acid metabolism, autophagy, glucagon and insulin signaling pathway, metabolic process and biological regulation.

**Supplementary Information:**

The online version contains supplementary material available at 10.1038/s41598-024-77910-9.

## Introduction

Large-scale epidemiological studies have revealed that vitamin D (VD) deficiency is prevalent worldwide^1^. VD is a kind of fat-soluble vitamin which mainly regulates calcium and phosphorus metabolism and maintain bone health. In recent years, its role in exoskeleton has received extensive attention. With the discovery of VD receptor (VDR) in fat cells, the relationship between VD and obesity has received increasing attention^2^.

Obesity is one of the most popular chronic diseases worldwide which leads to global burden on society. It is estimated that obesity will affect 60% of adult men, 50% of adult women, and 25% of children by 2050^3^. Our previous study was a large population-based cross-sectional multicenter study in which 5289 children aged 0–5 years were recruited from 12 Children’s Health Care Centers by a stratified cluster random-sampling method in 10 cities of Jiangsu Province, China. The results showed that the prevalence of VD deficiency in 0–5 years children was 30.1%^4^, and the prevalence of VD deficiency in 0–5 years children with obesity and non-obesity was 36.0%, 29.8%, respectively, there were all significant difference^5^. Vitamin D deficiency was observed in children with greater adiposity during the first 5 years of life. However, the mehcanism has not been well-defined.

Transfer RNA-derived small RNAs (tsRNAs) is a fragment derived from tRNA. According to the length and cleavage site of tRNA, tsRNA can be divided into two categories: tRNA-derived stress-induced tRNA fragments (tiRNAs), which are stress-induced mature tRNA fragments generated by the specific cleavage of 28-36-nucleotide (nt) anticodon ring, and transfer RNA-derived fragments (tRFs), which are derived from mature tRNAs or precursor tRNAs and are generated by specific cleavage of 14-30-nt-nucleotide anticodon rings^6,7^. White adipose tissue (WAT) in the mammalian body can be divided into subcutaneous adipose tissue (SAT) and visceral adipose tissue (VAT), which helps store energy, keep warm and protect the internal organs. In recent years, the dysregulation of tsRNA has gradually been a hotspot in clinical medicine, especially in the filed of cancer diseases^8,9^. However, tsRNAs participating in WAT of VD deficiency young male mice with or without obesity has been rarely reported. In this study, we analyzed the tsRNA expression profiles by high-throughput RNA sequencing to explore the new molecular mechanism of VD deficiency in regulating obesity.

## Materials and methods

### Establishment of animal models

12 healthy male C57BL/6J mice aged 4–6 weeks from Jiangsu Laboratory Animal Center in China were used in the study. To avoid interference with skin synthesis of vitamin D, a non-ultraviolet light source was used in the room, i.e. the ultraviolet light at 290–315 nm was filtered out. The animals were allowed free access to food and water at all times and were maintained on a 12 h light/dark cycle in a controlled temperature (20–25 °C) and humidity (50 ± 5%) environment. All animal experiments took place at Jiangsu Province Hospital Core Facility Center. All experimental procedures were reviewed and approved by Institutional Animal Care and Use Committee at Nanjing Medical University (IACUC-2112045). All procedures on animals followed the guidelines for humane treatment set by the Association of Laboratory Animal Sciences and the Center for Laboratory Animal Sciences at Nanjing Medical University. After one week of acclimatization, they were divided into 4 equal experimental groups according to the complete randomization. The ConVDS group was fed a standard purified diet (AIN93M) with 10% fat energy and 1000 IU/3606 kcal VD_3_. The ConVDD group was fed with low fat VD_3_ deficiency diet, the fat energy supply was 10%, and the VD_3_ content decreased to 0–25 IU/3606 kcal. HFVDS group was fed with normal diet with high fat VD_3_ content, fat supply energy reached 60% and VD_3_ content was 1000 IU/3606 kcal. In the HFVDD group, the fat supply reached 60% and the VD_3_ content was reduced to 0–25 IU/3606 kcal (Table S1). Diets used in HFVDD, HFVDS and ConVDD groups were adjusted appropriately on the basis of standard purified diets. All diets were purchased from TROPHIC Animal Feed High-tech Co. Ltd (Nantong, Jiangsu, China). Body mass and feed intakes were measured weekly. After 8 weeks, the body weight of HFVDD group was more than 20% higher than that of ConVDS group, indicating that the obesity animal model was successfully established. Three mice in the above groups were randomly selected, and 0.2 ml of blood was collected by removing eyeballs. VD is measured by the level of 25(OH)D in serum in the samples^10^. We measured the serum 25(OH)D levels using a Radioimmunoassay (RIA) kit (DiaSorin) with 125I labeled 25(OH)D as a tracer. The VD_3_ level in HFVDD group was lower than 80% of that in ConVDS group, suggesting that the model of obese mice with vitamin D deficiency was successfully established (Table S2).

### Samples preparation and RNA isolation

At week 8, all 12 mice were euthanased by cervical dislocation. The subcutaneous white adipose tissue (WAT) of each sample was collected in EDTA tubes and stored at − 80 °C for subsequent analysis. Three pieces (approximately 1000 mg each) of WAT from each sample were utilized for high-throughput sequencing assays, and another three pieces (approximately 100 mg each) of WAT from each sample were utilized for qRT-PCR assay. Total RNA was isolated using TRIzol (Invitrogen) according to the manufacturer’s instructions. RNA quantity was measured by using NanoDrop ND-1000 spectrophotometer and RNA integrity (quality) was assessed by Bioanalyzer 2100 or denaturing gel electrophoresis.

### RNA labeling and array hybridization

For small RNA microarray profiling, 100 ng of total RNA was first dephosphorylated with 3 units of T4 polynucleotide kinase (T4 PNK) at 37°C for 40 minutes to remove both (P) and (cP) chemical groups from the 3’ end of the RNAs, resulting in the formation of a 3-OH end. The reaction was terminated by heating to 70 °C for 5 min and then cooled immediately to 0 °C. Seven microliters of DMSO were added, and the mixture was heated to 100 °C for 3 min to denature the RNAs, followed by immediate cooling to 0 °C. RNA end labeling was conducted by adding ligase buffer, BSA, a final concentration of 50 mM pCp-Cy3, and 15 units of T4 RNA ligase in a 28 µL reaction at 16 °C overnight. The labeled reaction was then mixed with 22.5 µL of 2× hybridization buffer (Agilent) to achieve a final volume of 45 µL. This mixture was heated at 100 °C for 5 min and then cooled immediately to 0 °C. The 45 µL labeled sample mix was hybridized onto a microarray at 55 °C for 20 h. The slides were washed at room temperature in 6× SSC with 0.005% Triton X-102 for 10 min, followed by a wash in 0.1× SSC with 0.005% Triton X-102 for 5 min. Finally, the slides were scanned using an Agilent G2505C microarray scanner.

### Data analysis

Agilent Feature Extraction software (version 11.0.1.1) was used to analyze the acquired array images. The raw intensities were log2-transformed and quantile normalized. After normalization, probe signals with Present (P) or Marginal (M) QC flags above a certain threshold were retained. Multiple probes corresponding to the tsRNAs were averaged and consolidated into a single RNA level. Differentially expressed tsRNAs between the two comparison groups were identified by applying filters based on the specified fold change (FC) and statistical significance (p-value) thresholds. Volcano plots were generated using R software.

### Quantitative real time polymerase chain reaction (qRT-PCR) assay

Total RNA was extracted using TRIzol (Invitrogen, USA), and cDNA was synthesized using the Evo M-MLV First Strand cDNA Synthesis Kit (AG11711, Accurate Biology). Amplification was performed using the SYBR Green Premix Pro Taq HS qPCR Kit (AG11701, Accurate Biology) on a StepOnePlus (Applied Biosystems) system, following the manufacturer’s protocol. U6 was used as the reference gene to normalize the cycle threshold (CT) values, and relative expression levels of tRNAs were calculated using the 2^−△△CT^ method. At least three technical replicates were performed for each sample and gene to ensure accurate quantification. The primer sequences used in this study were designed and synthesized by RiboBio (Guangzhou, China) and Aiji (Guangzhou, China).

### GO analysis

The Gene Ontology project offers a standardized vocabulary for describing the characteristics of genes and their products across various organisms (http://www.geneontology.org). This ontology includes three primary domains: Biological Process, Cellular Component, and Molecular Function. To assess whether the overlap between the list of differentially expressed (DE) genes and the Gene Ontology (GO) annotation list is greater than what would be expected by chance, Fisher’s exact test, available in Bioconductor’s topGO package, is utilized. The p-value generated by topGO reflects the significance of GO term enrichment within the DE genes, with lower p-values indicating greater significance for the GO terms (a p-value of ≤ 0.05 is generally recommended).

### Pathway analysis

Pathway analysis involves a functional assessment that links genes to KEGG pathways^11–13^. The p-value (which can be represented as the EASE score, Fisher’s p-value, or hypergeometric p-value) indicates the significance of a given pathway in relation to the conditions being studied. A lower p-value signifies a more significant pathway, with a recommended cut-off of 1.0.

### Prediction of target genes of DE tRFs

The target gene prediction method utilized a combination of the miRanda and TargetScan algorithms for local target predictions based on sequence analysis. The miRanda algorithm is capable of identifying any seed type site, employing a dynamic programming approach that considers RNA secondary structures and free energy. Target genes were chosen based on high structure scores and low free energy. Meanwhile, TargetScan leveraged mRNA and tRF expression profile data to identify biologically significant sites using relatively conservative scoring models. Genes with low context scores were selected through this process. By integrating the miRanda and TargetScan algorithms, the strengths of both approaches were combined, enhancing the presentation of results. Genes that exhibited overlapping results from miRanda (structure score ≥ 140 and energy ≤-9) and TargetScan (context ≤-0.1) were regarded as potential targets of tRFs. The top 20 target genes and differential tsRNA were then selected to construct a co-expression network, which was visualized using Cytoscape 3.6.1 software.

## Results

### Characterization and expression profile of the extracted RNAs

For spectrophotometer, the OD A260/A280 ratios of the samples vary from 1.87 to 1.99, and the OD A260/A230 ratios range from 1.85 to 2.41. All samples had sufficient sequence counts and quality for the following analysis (Table S3). RNA Integrity and gDNA contamination were tested by Denaturing Agarose Gel Electrophoresis (Figure S1). Based on the strict quality control, a total of 1598 tsRNAs were identified in the subcutaneous adipose tissue samples.

### Differentially expressed tsRNAs identified in mice subcutaneous adipose tissue

**Figure 1:**
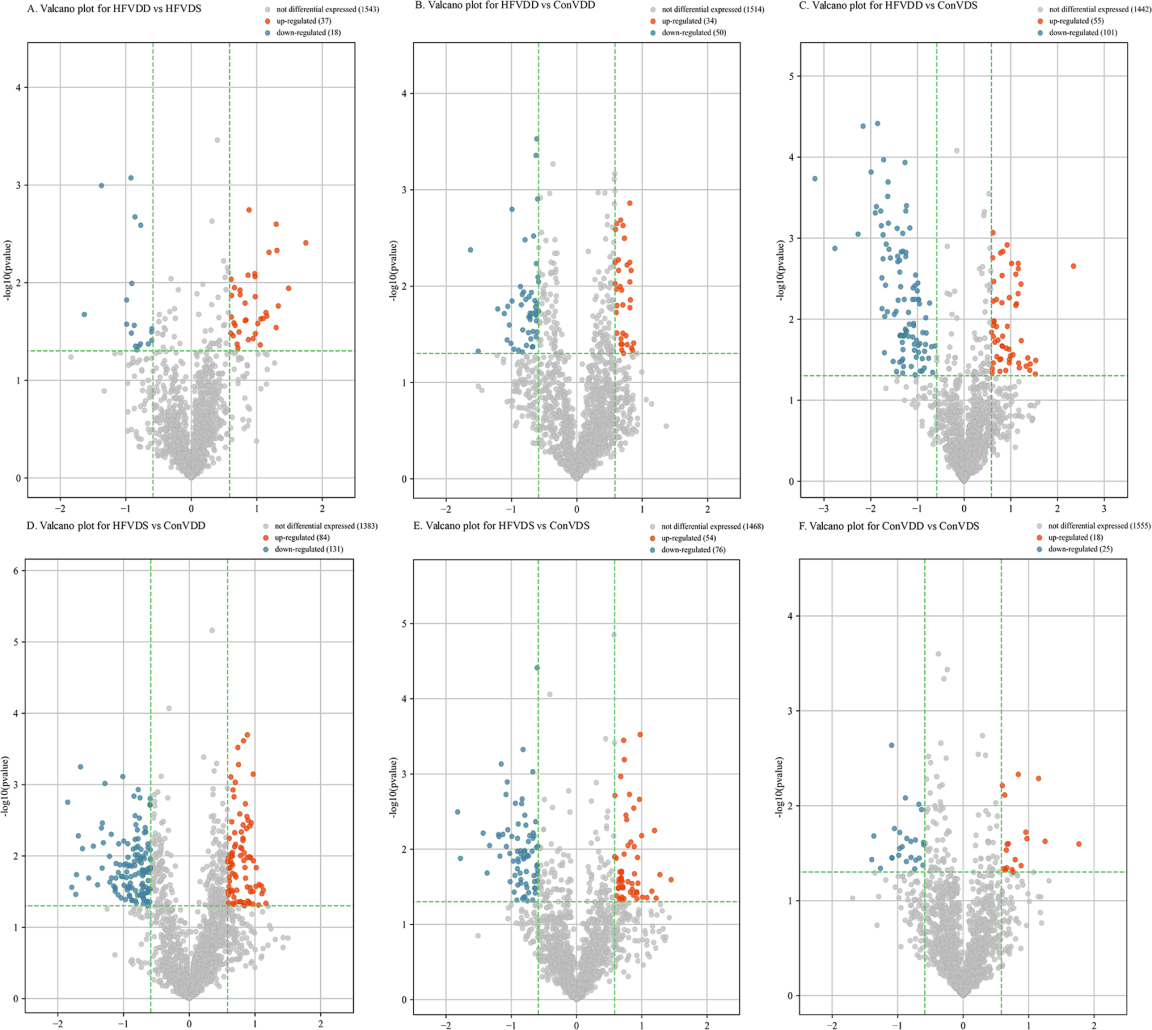
Valcano plot. **A**: Valcano plot for HDVDD vs. HFVDS; **B**: Valcano plot for HFVDD vs. ConVDD; **C**: Valcano plot for HFVDD vs. ConVDS; **D**: Valcano plot for HFVDS vs. ConVDD; **E**: Valcano plot for HFVDS vs. ConVDS; F Valcano plot for ConVDD vs. ConVDS.

To further explore the differentially expressed tsRNAs in the four groups, we performed pairwise comparisons among the samples, and the identification criteria of differential expression were a fold change (FC) ≥ 1.5 or ≤ 0.67 (*p* < 0.05). As was shown in Table S4, a total of 37 up-regulated tsRNAs and 18 tsRNAs were identified in HFVDD vs. HFVDS (Fig. 1A). 34 up-regulated tsRNAs and 50 down-regulated tsRNAs were identified in HFVDD vs. ConVDD (Fig. 1B). 55 up-regulated tsRNAs and 101 down-regulated tsRNAs were identified in HFVDD vs. ConVDS (Fig. 1C). 84 up-regulated tsRNAs and 131 down-regulated tsRNAs were identified in HFVDS vs. ConVDD (Fig. 1D). 54 up-regulated tsRNAs and 76 down-regulated tsRNAs were identified in HFVDS vs. ConVDS (Fig. 1E). 18 up-regulated tsRNAs and 25 down-regulated tsRNAs were identified in ConVDD vs. ConVDS (Fig. 1F).

### Between-group comparisons of the differentially expressed tsRNAs in VD deficiency mice with or without obesity by high-throughput sequencing technology

We listed the top 10 tsRNAs with the most significantly differential expression in each group according to the FCs (Table 1). 5 tsRNAs (tRF5-20-HisGTG-3, tRF5-22-CysGCA-27, tRF5-20-CysGCA-27, 5’tiRNA-36-ValAAC-3, tRF5-18-CysGCA-27) were highly expressed and 10 tsRNAs (mt-5’tiRNA-33-AlaTGC, mt-5’tiRNA-33-SerTGA, mt-5’tiRNA-32-SerTGA and mt-tRF3a-ProTGG, mt-5’tiRNA-38-LeuTAA, mt-5’tiRNA-31-GluTTC, mt-5’tiRNA-36-LeuTAA, mt-tRF3b-TrpTCA, mt-5’tiRNA-37-LeuTAA, mt-5’tiRNA-32-TrpTCA) were low expressed not only in HFVDD vs. HFVDS, but aslo in HFVDD vs. ConVDS. 5 tsRNAs (tRF3a-GlyGCC-1, 3’tiRNA-50-SerGCT-1, tRF3b-SerGCT-1, tRF3a-ThrAGT-5, 3’tiRNA-41-GlyGCC-6) were highly expressed only in HFVDD vs. ConVDS.

**Table 1:** Part of the significantly differentially expressed tsRNAs in each group (sorted by FC).

Group	tsRNA ID	type	tsRNA-sequence	FC
HFVDD vs. HFVDS (up regulated)				
	tRF5-20-HisGTG-3	5’tRF	GCCGTGATCGTATAGGGGTT	3.36
	tRF3a-GlyGCC-1	3’tRF	TTCCCGGCCCATGCACCA	2.80
	tRF5-22-CysGCA-27	5’tRF	GCGGGTATAGCTCAGGGGTAGA	2.51
	3’tiRNA-50-SerGCT-1	3’tiRNA	TAATCCATTGTGCTCTGCACGCGTGGGTTCGAATCCCACCTTCGTCGCCA	2.48
	tRF3b-SerGCT-1	3’tRF	TCGAATCCCACCTTCGTCGCCA	2.46
	tRF5-20-CysGCA-27	5’tRF	GCGGGTATAGCTCAGGGGTA	2.46
	tRF3a-ThrAGT-5	3’tRF	ATCCCAGCAAGGCCTCCA	2.28
	5’tiRNA-36-ValAAC-3	5’tiRNA	GTTTCCGTAGTGTAGTGGTCATCACGCTCGCCTAAC	2.23
	3’tiRNA-41-GlyGCC-6	3’tiRNA	CATGAGGGAGGCCCAGGTTCAATTCCAGGCCCATTGCACCA	2.20
	tRF5-18-CysGCA-27	5’tRF	GCGGGTATAGCTCAGGGG	2.14
HFVDD vs. HFVDS (down regulated)				
	mt-5’tiRNA-33-AlaTGC	5’tiRNA	GAGGTCTTAGCTTAATTAAAGCAATTGATTTGC	0.32
	mt-5’tiRNA-33-SerTGA	5’tiRNA	GAGAAAGACATATAGGATATGAGATTGGCTTGA	0.39
	mt-5’tiRNA-32-SerTGA	5’tiRNA	GAGAAAGACATATAGGATATGAGATTGGCTTG	0.50
	mt-tRF3a-ProTGG	3’tRF	GCTCCTTCTTCTTGACCA	0.50
	mt-5’tiRNA-38-LeuTAA	5’tiRNA	ATTAGGGTGGCAGAGCCAGGAAATTGCGTAAGACTTAA	0.53
	mt-5’tiRNA-31-GluTTC	5’tiRNA	GTTTCTGTAGTTGAATTACAACGATGATTTT	0.53
	mt-5’tiRNA-36-LeuTAA	5’tiRNA	ATTAGGGTGGCAGAGCCAGGAAATTGCGTAAGACTT	0.53
	mt-tRF3b-TrpTCA	3’tRF	CACACAAGTTTAACTTCTGCCA	0.55
	mt-5’tiRNA-37-LeuTAA	5’tiRNA	ATTAGGGTGGCAGAGCCAGGAAATTGCGTAAGACTTA	0.55
	mt-5’tiRNA-32-TrpTCA	5’tiRNA	AGAAGTTTAGGATATACTAGTCCGCGAGCCTT	0.55
HFVDD vs. ConVDD (up regulated)				
	mt-tRF5-22-LeuTAA	5’tRF	ATTAGGGTGGCAGAGCCAGGAA	1.83
	tRF5-20-SerCGA-2	5’tRF	GTCACGGTGGCCGAGTGGTT	1.81
	5’tiRNA-34-GlyCCC-1	5’tiRNA	GCGCCGCTGGTGTAGTGGTATCATGCAAGATTCC	1.79
	5’tiRNA-35-AsnGTT-1	5’tiRNA	GTCTCCGTGGCGCAATCGGTCAGCGCGTTCGGCTG	1.78
	5’tiRNA-37-LeuTAG-1	5’tiRNA	GGTAGCGTGGCCGAGCGGTCTAAGGCGCTGGATTTAG	1.76
	tRF5-18-ArgTCT-2	5’tRF	GGCTCTGTGGCGCAATGG	1.76
	tRF5-20-HisGTG-2	5’tRF	GCCGTGATCGTATAGTGGTT	1.75
	5’tiRNA-35-AsnGTT-2	5’tiRNA	GTCTCTGTGGCGCAATTGGTTAGCGCGTTCGGCTG	1.75
	tRF5-22-HisGTG-2	5’tRF	GCCGTGATCGTATAGTGGTTAG	1.75
	tRF5-18-ProAGG-3	5’tRF	AGCTCATTAGTCTAGGGG	1.71
HFVDD vs. ConVDD (down regulated)				
	tRF5-18-iMetCAT-1	5’tRF	AGCAGAGTGGCGCAGCGG	0.32
	tRF5-22-AlaAGC-19	5’tRF	GTTGGAGATTTAGCTCAGCGGT	0.35
	tRF5-18-TrpCCA-3	5’tRF	GGCCTCGTGGCGCAACGG	0.43
	mt-tRF3a-TyrGTA	3’tRF	ATCCTCTTTTTACCACCA	0.46
	mt-tRF3b-TyrGTA	3’tRF	TTAAATCCTCTTTTTACCACCA	0.48
	mt-3’tiRNA-39-HisGTG	3’tiRNA	GAATCTGACAACAGGAAATAAACCTCCTTATTCACCCCA	0.48
	mt-tRF3a-IleGAT	3’tRF	GCCCTCTTATTTCTACCA	0.49
	mt-tRF3b-IleGAT	3’tRF	TCAAGCCCTCTTATTTCTACCA	0.50
	tRF5-18-AlaTGC-5	5’tRF	GGGGATGTAGCTCAGCGG	0.50
	tRF5-18-ValCAC-5	5’tRF	GTTTTTGTAGTGTAGCGG	0.50
HFVDD vs. ConVDS (up regulated)				
	tRF5-20-HisGTG-3	5’tRF	GCCGTGATCGTATAGGGGTT	5.07
	tRF5-22-CysGCA-27	5’tRF	GCGGGTATAGCTCAGGGGTAGA	2.89
	tRF5-20-CysGCA-27	5’tRF	GCGGGTATAGCTCAGGGGTA	2.88
	tRF5-20-LysTTT-1	5’tRF	GCCCGGATAGCTCAGTCGGT	2.66
	5’tiRNA-34-ValAAC-3	5’tiRNA	GTTTCCGTAGTGTAGTGGTCATCACGCTCGCCTA	2.65
	tRF5-18-CysGCA-27	5’tRF	GCGGGTATAGCTCAGGGG	2.57
	tRF5-18-CysGCA-17	5’tRF	TGGGGTATAGCTCAGGGG	2.51
	5’tiRNA-35-ValAAC-3	5’tiRNA	GTTTCCGTAGTGTAGTGGTCATCACGCTCGCCTAA	2.34
	tRF5-18-HisGTG-2	5’tRF	GCCGTGATCGTATAGTGG	2.33
	tRF5-22-CysGCA-17	5’tiRNA	GTTTCCGTAGTGTAGTGGTCATCACGCTCGCCTCA	2.25
HFVDD vs. ConVDS (down regulated)				
	mt-5’tiRNA-33-SerTGA	5’tiRNA	GAGAAAGACATATAGGATATGAGATTGGCTTGA	0.11
	mt-5’tiRNA-32-SerTGA	5’tiRNA	GAGAAAGACATATAGGATATGAGATTGGCTTG	0.15
	mt-5’tiRNA-33-AlaTGC	5’tiRNA	GAGGTCTTAGCTTAATTAAAGCAATTGATTTGC	0.21
	mt-tRF3a-ProTGG	3’tRF	GCTCCTTCTTCTTGACCA	0.22
	mt-tRF3b-IleGAT	3’tRF	TCAAGCCCTCTTATTTCTACCA	0.25
	mt-tRF3a-AspGTC	3’tRF	AATCTATATATCTTACCA	0.27
	mt-tRF3b-ProTGG	3’tRF	AGTAGCTCCTTCTTCTTGACCA	0.27
	mt-tRF3a-IleGAT	3’tRF	GCCCTCTTATTTCTACCA	0.28
	mt-tRF3a-TyrGTA	3’tRF	ATCCTCTTTTTACCACCA	0.29
	mt-5’tiRNA-30-CysGCA	5’tiRNA	GGTCTTAAGGTGATATTCATGTCGAATTGC	0.29
HFVDS vs. ConVDD (up regulated)				
	5’tiRNA-37-LeuTAG-1	5’tiRNA	GGTAGCGTGGCCGAGCGGTCTAAGGCGCTGGATTTAG	2.25
	5’tiRNA-34-AlaAGC-4	5’tiRNA	GGGGGATTAGCTCAAATGGTAGAGCGCTCGCTTA	2.19
	5’tiRNA-34-AlaAGC-6	5’tiRNA	GGGGAATTAGCTCAAATGGTAGAGCGCTCGCTTA	2.15
	5’tiRNA-33-ProTGG-1	5’tiRNA	GGCTCGTTGGTCTAGGGGTATGATTCTCGGTTT	2.14
	5’tiRNA-33-ProAGG-2	5’tiRNA	GGCTTGTTGGTCTAGGGGTATGATTCTCACTTA	2.09
	5’tiRNA-34-GlyCCC-1	5’tiRNA	GCGCCGCTGGTGTAGTGGTATCATGCAAGATTCC	2.08
	tRF5-31-GlyGCC-1	5’tRF	GCATGGGTGGTTCAGTGGTAGAATTCTCGCC	2.03
	5’tiRNA-33-ProTGG-3	5’tiRNA	GGTTTGTTGGTCTAGTGGTATGATTCTCAGTTT	2.02
	5’tiRNA-33-ProTGG-2	5’tiRNA	GGCTCGTTGGTCTAGGGGTATGATTCTCGCTTT	1.97
	tRF5-18-AlaCGC-5	5’tRF	TCCCTGGTAGTCTAGTGG	1.96
HFVDS vs. ConVDD (down regulated)				
	tRF5-18-iMetCAT-1	5’tRF	AGCAGAGTGGCGCAGCGG	0.28
	tRF3a-MetCAT-4	3’tRF	GCCTCAGAGAAGGCACCA	0.29
	tRF3a-GlyGCC-1	3’tRF	TTCCCGGCCCATGCACCA	0.30
	tRF3a-ThrAGT-5	3’tRF	ATCCCAGCAAGGCCTCCA	0.30
	tRF5-22-AlaAGC-19	5’tRF	GTTGGAGATTTAGCTCAGCGGT	0.31
	tRF5-20-LysCTT-18	5’tRF	ACCCAGCTAGTTCAGTCGGT	0.32
	tRF3a-LysTTT-3	3’tRF	GTCCCTGTTCAGGCGCCA	0.32
	3’tiRNA-41-GlyGCC-6	3’tiRNA	CATGAGGGAGGCCCAGGTTCAATTCCAGGCCCATTGCACCA	0.35
	tRF3a-AlaAGC-1	3’tRF	TCCCCAGCACCTCCACCA	0.36
	tRF3b-MetCAT-4	3’tRF	TCGAGCCTCAGAGAAGGCACCA	0.38
HFVDS vs. ConVDS (up regulated)				
	tRF5-20-AlaAGC-6	5’tRF	GGGGGTATAGCTCAGTGG	2.73
	tRF5-20-AlaAGC-10	5’tRF	GTAGTCGTGGCCGAGTGGTTAAGG	2.43
	5’tiRNA-33-ProTGG-4	5’tRF	TCCCTGGTGGTCTAGTGGCT	2.33
	tRF5-18-AlaCGC-5	5’tiRNA	GGCTCGTTGGTCTAGGGGTATGATTCTCACTTT	2.29
	tRF5-18-AlaAGC-10	5’tRF	GCATTGGTAGTTCAATGGTAGA	2.23
	5’tiRNA-33-ProAGG-2	5’tiRNA	GACCTCGTGGCACAATGGTAGCACGTCTGACTC	2.11
	5’tiRNA-34-ArgACG-1	5’tRF	GGCTCGTTGGTCTAGGGGTATG	2.02
	tRF5-22-ArgCCG-3	5’tRF	GCCCGGATAGCTCAGTCGGT	2.00
	tRF5-20-ArgCCG-3	5’tiRNA	GGGGATGTAGCTCAGCGGTAGAGCACATGCTTT	1.97
	tRF5-20-LysTTT-1	5’tiRNA	GGCCGGTTAGCTCAGTTGGTTAGAGCATGGTGCTAA	1.95
HFVDS vs. ConVDS (down regulated)				
	mt-5’tiRNA-33-SerTGA	5’tiRNA	GAGAAAGACATATAGGATATGAGATTGGCTTGA	0.28
	mt-5’tiRNA-32-SerTGA	5’tiRNA	GAGAAAGACATATAGGATATGAGATTGGCTTG	0.29
	tRF3a-ThrAGT-5	3’tRF	ATCCCAGCAAGGCCTCCA	0.37
	tRF3a-GlyGCC-1	3’tRF	TTCCCGGCCCATGCACCA	0.39
	mt-tRF3b-IleGAT	3’tRF	TCAAGCCCTCTTATTTCTACCA	0.40
	mt-tRF3a-IleGAT	3’tRF	GCCCTCTTATTTCTACCA	0.44
	mt-tRF3b-ProTGG	3’tRF	AGTAGCTCCTTCTTCTTGACCA	0.44
	mt-tRF3a-ProTGG	3’tRF	GCTCCTTCTTCTTGACCA	0.44
	mt-3’tiRNA-39-HisGTG	3’tiRNA	GAATCTGACAACAGGAAATAAACCTCCTTATTCACCCCA	0.45
	mt-tRF3a-AspGTC	3’tRF	AATCTATATATCTTACCA	0.46
ConVDD vs. ConVDS (up regulated)				
	tRF5-18-iMetCAT-1	5’tRF	AGCAGAGTGGCGCAGCGG	3.41
	tRF5-20-LysCTT-18	5’tRF	ACCCAGCTAGTTCAGTCGGT	2.38
	tRF5-18-TrpCCA-3	5’tRF	GGCCTCGTGGCGCAACGG	2.22
	tRF5-18-AlaTGC-5	5’tRF	GGGGATGTAGCTCAGCGG	1.96
	tRF5-18-ValCAC-5	5’tRF	GTTTTTGTAGTGTAGCGG	1.94
	tRF3b-LeuTAA-5	3’tRF	TCAAAGCTTGGACGAGTCCCCA	1.85
	tRF5-20-LysTTT-2	5’tRF	GCCTGGATAGCTCAGTCGGT	1.79
	3’tiRNA-41-MetCAT-4	3’tiRNA	TAATCTGAAGGTCCTGAGTTCGAGCCTCAGAGAAGGCACCA	1.74
	3’tiRNA-41-LysCTT-7	3’tiRNA	TAATCCCAGGGTCATGGGTTCGAGCCCCATATTAGGCACCA	1.70
	tRF3a-LeuTAA-5	3’tRF	AGCTTGGACGAGTCCCCA	1.68
ConVDD vs. ConVDS (down regulated)				
	mt-5’tiRNA-30-CysGCA	5’tiRNA	GGTCTTAAGGTGATATTCATGTCGAATTGC	0.38
	5’tiRNA-35-GlyGCC-5	5’tiRNA	GCATTGGTGGTTCAGTGGTAGAATTCTTGCCTGCC	0.39
	mt-5’tiRNA-21-SerGCT	5’tiRNA	AAGAAAGATTGCAAGAACTGC	0.42
	5’tiRNA-33-GlyGCC-1	5’tiRNA	GCATGGGTGGTTCAGTGGTAGAATTCTCGCCTG	0.47
	mt-tRF3b-ProTGG	3’tRF	AGTAGCTCCTTCTTCTTGACCA	0.47
	5’tiRNA-35-GlyGCC-1	5’tiRNA	GCATGGGTGGTTCAGTGGTAGAATTCTCGCCTGCC	0.47
	5’tiRNA-35-GlyGCC-6	5’tiRNA	GCATGGGTGGTTCAGTGGTAGAATTCTCACCTGCC	0.48
	mt-tRF3b-IleGAT	3’tRF	TCAAGCCCTCTTATTTCTACCA	0.51
	5’tiRNA-34-GlyGCC-1	5’tiRNA	GCATGGGTGGTTCAGTGGTAGAATTCTCGCCTGC	0.51
	5’tiRNA-36-GluCTC-6	5’tiRNA	TCCCTGGCGGCCTAGTGGTTAGGATTCAGTGCTCTC	0.51

### Between-group comparisons of the selected differentially expressed tsRNAs in VD deficiency mice with or without obesity verified by RT-PCR

**Figure 2:**
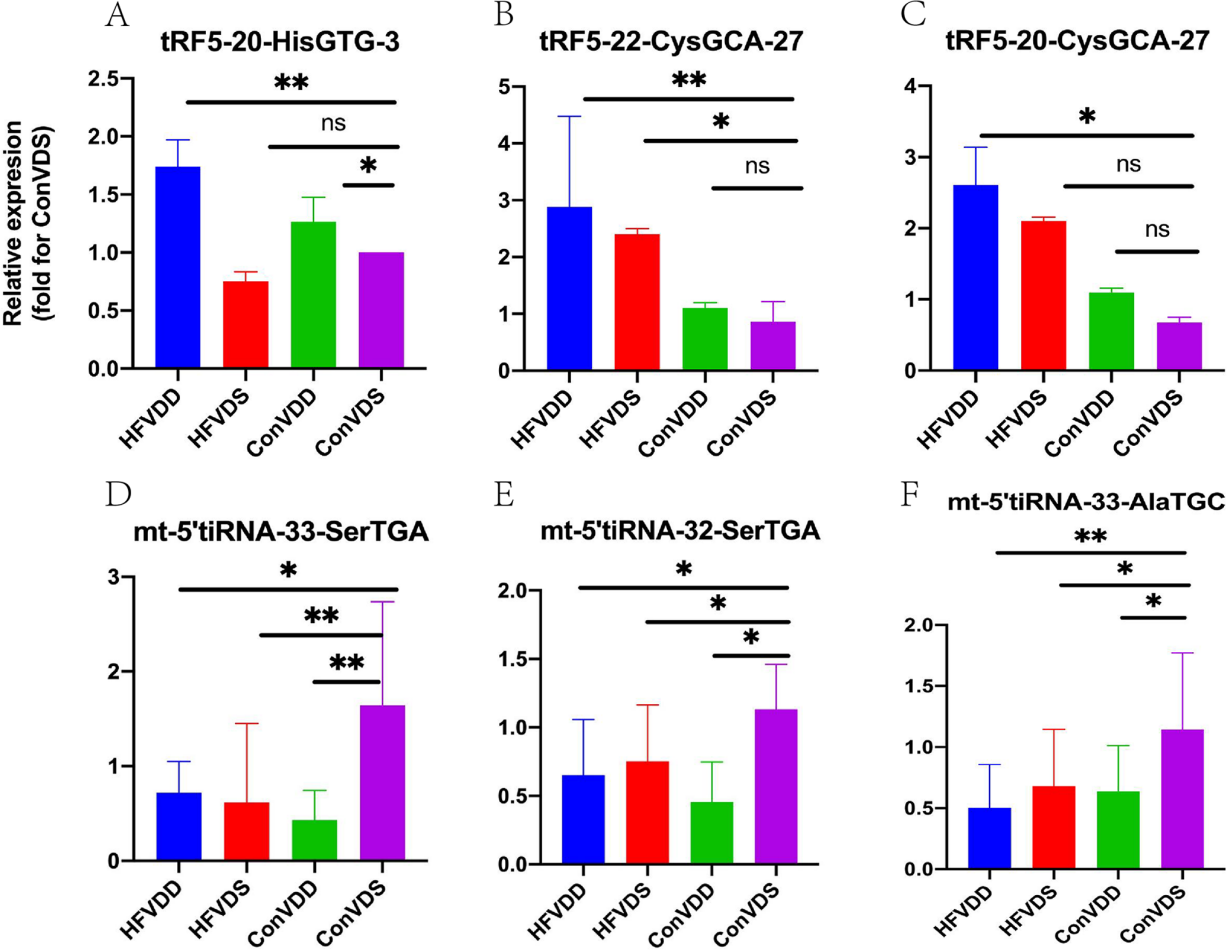
Between-group comparisons of the selected differentially expressed tsRNAs in VD deficiency mice with or without obesity verified by RT-PCR. **A**: tRF5-20-HisGTG-3; **B**: tRF5-22-CysGCA-27; **C**: mt-5_tiRNA-33-AlaTGC; **D**: mt-5_tiRNA-33-SerTGA; **E**: mt-5_tiRNA-32-SerTGA; **F**:mt-tRF3a-ProTGG.

In the next step, in order to validate the differential expression, 2 high candidate tRFs (tRF5-20-HisGTG-3 and tRF5-22-CysGCA-27) and 4 low candidate tRFs (mt-5’tiRNA-33-AlaTGC, mt-5’tiRNA-33-SerTGA, mt-5’tiRNA-32-SerTGA and mt-tRF3a-ProTGG) not only in HFVDD vs. HFVDS, but aslo in HFVDD vs. ConVDS were measured by RT-PCR. 1 high candidate tRFs (tRF3a-GlyGCC-1) in HFVDD vs. ConVDS was measured by RT-PCR. RT-PCR verification further demonstrated that 1 tsRNAs (tRF5-20-HisGTG-3, *P* < 0.05, Fig. 2A) was significantly up-regulated and 1 tsRNA (mt-tRF3a-ProTGG, *P* < 0.05, Fig. 2F) was significantly down-regulated not only in HFVDD vs. HFVDS, but aslo in HFVDD vs. ConVDS. 1 tsRNAs (tRF5-22-CysGCA-27, *P* < 0.05, Fig. 2B) was significantly up-regulated and 3 tsRNA (mt-5’tiRNA-33-SerTGA, mt-5’tiRNA-32-SerTGA, and mt-5’tiRNA-33-AlaTGC, all *P* < 0.05, Fig. 2D, E and C) were significantly down-regulated only in HFVDD vs. ConVDS.

### GO enrichment, KEGG pathway analyses, and associated target genes of the candidate tsRNAs

**Figure 3:**
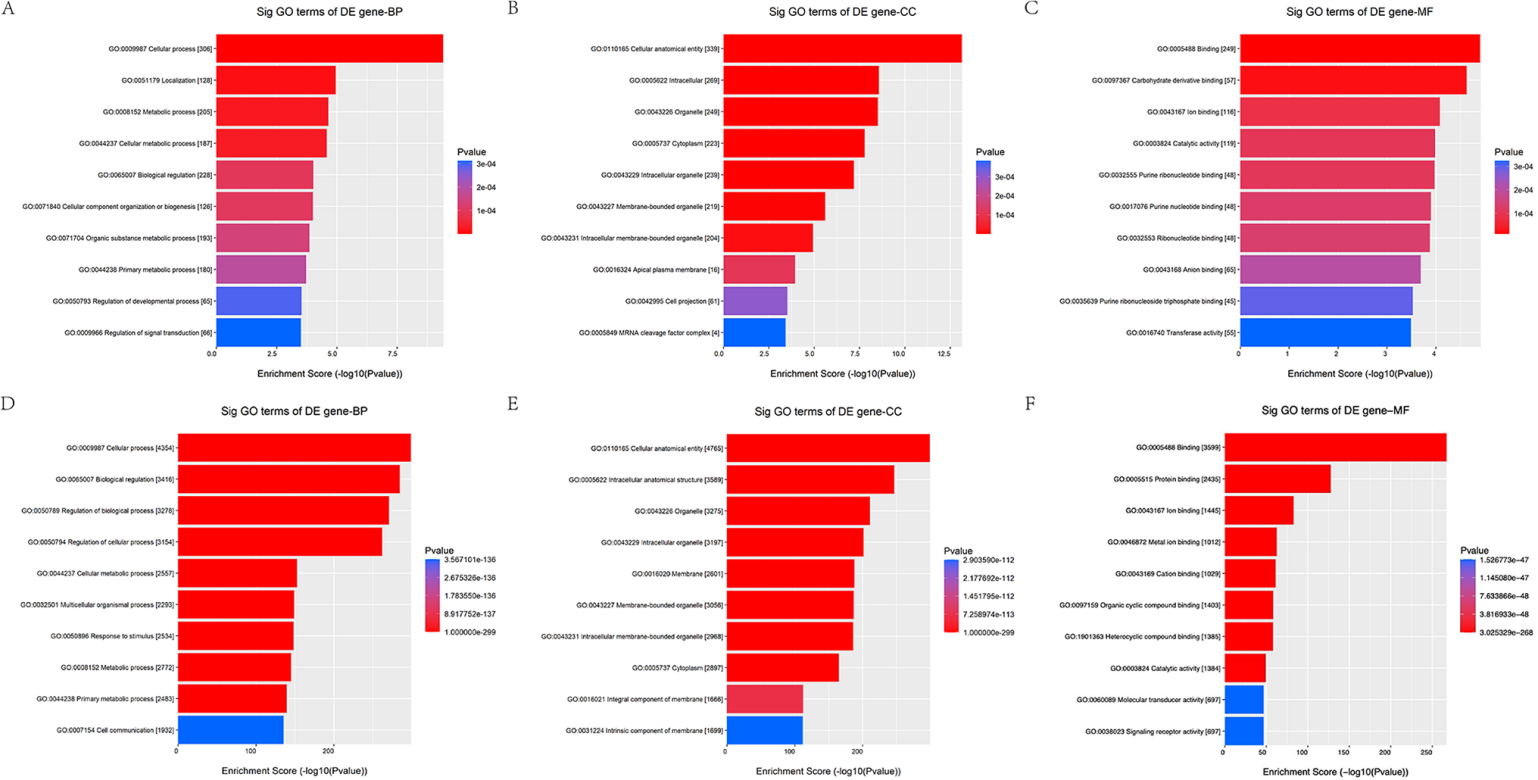
The BP, CC and HF of uptsRNA(**A**, **B**,**C**) and the down tsRNA(**D**, **E**,**F**). A: the BP of uptsRNA; B: the CC of uptsRNA; C: the HF of uptsRNA; D: the BP of the down tsRNA; E: the CC of the down tsRNA; F: the HF of the down tsRNA.

The functional annotation of candidate tsRNAs were performed as previously described. The biological processes (BP) terms including cellular process, biological regulation, cellular metabolic process, regulation of biological process, regulation of cellular process, metabolic process, primary metabolic process, organic substance metabolic process, nitrogen compound metabolic process, cellular macromolecule metabolic process were found to highly enriched in the target genes of 3 up-regulated tsRNAs (Fig. 3A). The BP terms including cellular process, biological regulation, regulation of biological process, regulation of cellular process, cellular metabolic process, multicellular organismal process, response to stimulus, metabolic process, primary metabolic process, cell communication were found to highly enriched in the target genes of 4 down-regulated tsRNAs (Fig. 3D). The cellular components (CC) terms including cellular anatomical entity, intracellular, organelle, intracellular organelle, membrane − bounded organelle, intracellular membrane − bounded organelle, cytoplasm, membrane, nucleus, intrinsic component of membrane were found to highly enriched in the target genes of 3 up-regulated tsRNAs (Fig. 3B). The CC terms including cellular anatomical entity, intracellular anatomical structure, organelle, intracellular organelle, membrane, membrane − bounded organelle, intracellular membrane − bounded organelle, cytoplasm, integral component of membrane, intrinsic component of membrane were found to highly enriched in the target genes of 4 down-regulated tsRNAs (Fig. 3E). The molecular functions (MF) terms including binding, protein binding, on binding, heterocyclic compound binding, organic cyclic compound binding, cation binding, metal ion binding, catalytic activity, enzyme binding, identical protein binding were found to highly enriched in the target genes of 3 up-regulated tsRNAs (Fig. 3C). The MF terms including binding, protein binding, ion binding, metal ion binding, cation binding, organic cyclic compound binding, heterocyclic compound binding, catalytic activity, molecular transducer activity, signaling receptor activity were found to highly enriched in the target genes of 4 down-regulated tsRNAs (Fig. 3F).

**Figure 4:**
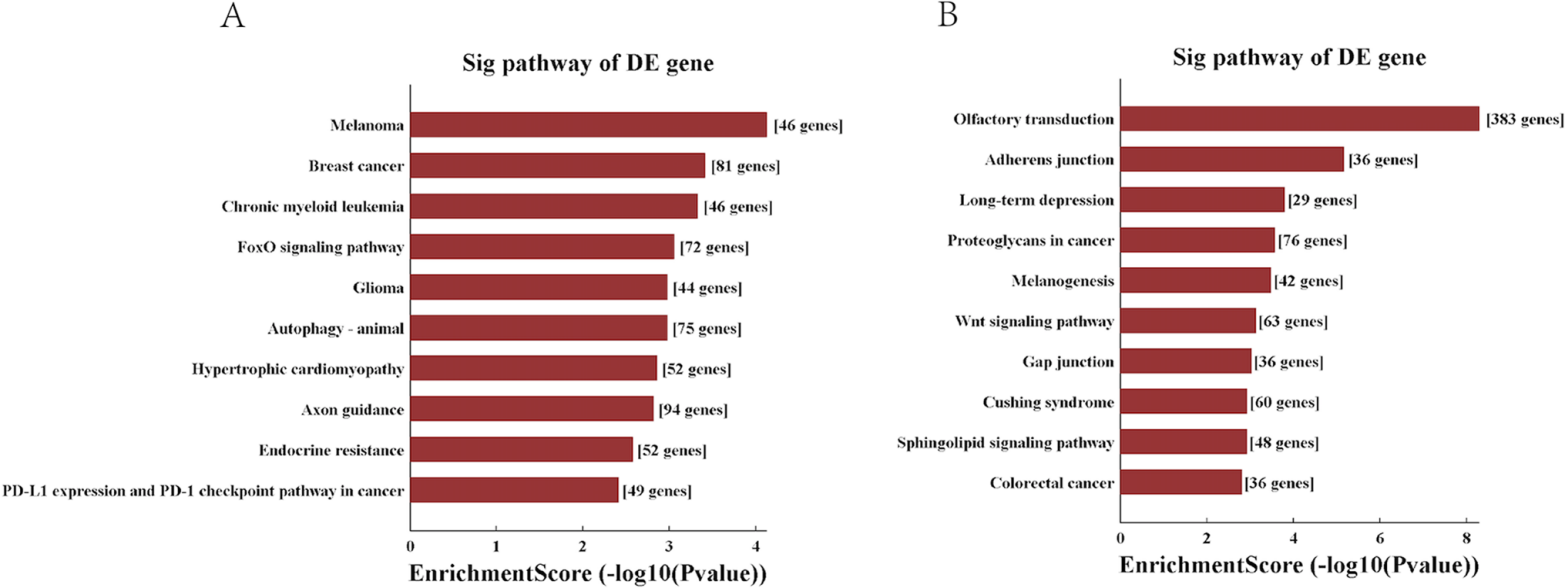
The Sig pathway of DE gene in the up tsRNA (**A**) and the down tsRNA(**B**). A: the Sig pathway of DE gene in the up tsRNA; B: the Sig pathway of DE gene in the down tsRNA;.

The top 10 significant pathways of candidate tsRNA were shown in Fig. 4. It was demonstrated that the 3 up-regulated tsRNAs seemed to be most strongly associated with melanoma, breast cancer, chronic myeloid leukemia, FoxO signaling pathway glioma, autophagy-animal, hypertrophic cardiomyopathy, axon guidance, endocrine resistance, PD-L1 expression and PD-1 checkpoint pathway in cancer (Fig. 4A). By contrast, 4 down-regulated tsRNAs seemed to be most strongly associated with olfactory transduction, colorectal cancer, sphingolipid signaling pathway, cushing syndrome, gap junction, wnt signaling pathway, melanogenesis, proteoglycans in cancer, long − term depression, adherens junction (Fig. 4B). 

**Figure 5:**
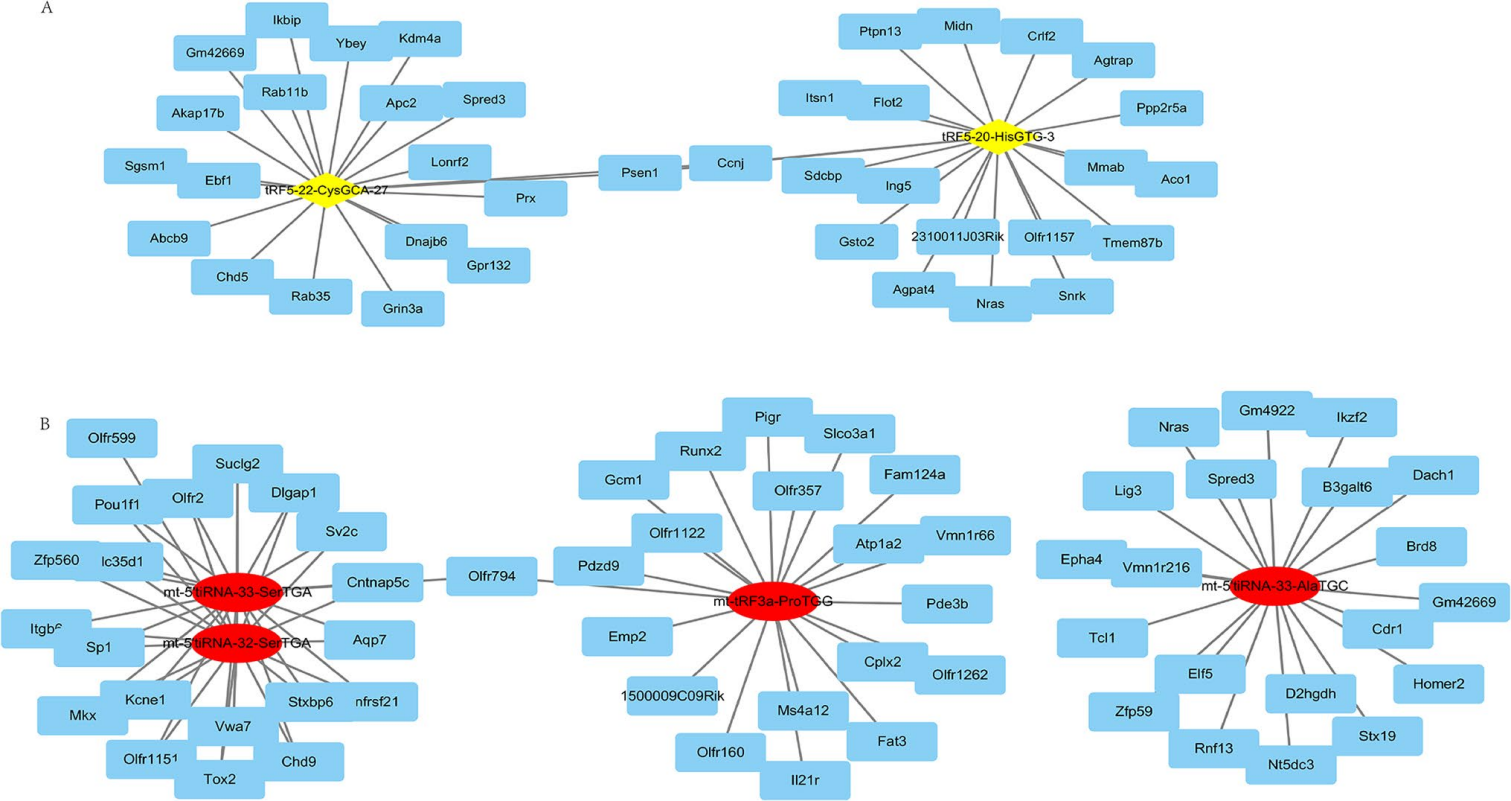
The co-expression network of the top 20 target genes and the up tsRNA(**A**) and the down tsRNA(**B**). A: The co-expression network of the top 20 target genes and the up tsRNA; B: The co-expression network of the top 20 target genes and the down tsRNA.

TargetScan and Miranda were used to predict the target genes of the qPCR-verified DE tsRNAs. There were 403, 330, 4182, 4425, 4324 and 2564 targeted genes of tRF5-20-HisGTG-3, tRF5-22-CysGCA-27, mt-5’tiRNA-33-AlaTGC, mt-5’tiRNA-33-SerTGA, mt-5’tiRNA-32-SerTGA and mt-tRF3a-ProTGG, respectively. The top 20 potential targeted genes of tRF5-20-HisGTG-3, tRF5-22-CysGCA-27, mt-5’tiRNA-33-AlaTGC, mt-5’tiRNA-33-SerTGA, mt-5’tiRNA-32-SerTGA and mt-tRF3a-ProTGG, respectively were listed in Table 2. The co-expression network of the top 20 target genes and differential tsRNA is shown in Fig. 5A and Fig. 5B.

**Table 2:** The top 20 predicted target genes of tsRNA.

tsRNA	The top 20 predicted target genes
tRF5-20-HisGTG-3	Tmem87b, Aco1, Gsto2, Ppp2r5a, Midn, Itsn1, Olfr1157, Ptpn13, Crlf2, Agpat4, Snrk, Sdcbp, Mmab, Agtrap, 2310011J03Rik, Flot2, Ing5, Imp4, 9930012K11Rik, Ubr7
tRF5-22-CysGCA-27	Akap17b, Chd5, Lonrf2, Grin3a, Ccnj, Ikbip, Rab11b, Abcb9, Prx, Sgsm1, Gpr132, Kdm4a, Ybey, Dnajb6, Rab35, Apc2, Smim24, Syndig1l, Cers3, Tfdp1
mt-5_tiRNA-33-AlaTGC	Nt5dc3, Cdr1, Dach1, B3galt6, Gm4922, Homer2, Lig3, Zfp59, D2hgdh, Brd8, Gm42669, Ikzf2, Epha4, Vmn1r216, Elf5, Tcl1, Rnf13, Stx19, Slc41a2, Tff3
mt-5_tiRNA-33-SerTGA	Pou1f1, Olfr2, Sp1, Tnfrsf21, Itgb6, Aqp7, Chd9, Suclg2, Vwa7, Mkx, Cntnap5c, Olfr1151, Sv2c, Zfp560, Kcne1, Stxbp6, Slc35d1, Tox2, Dlgap1, Fn3krp
mt-5_tiRNA-32-SerTGA	Pou1f1, Olfr2, Tnfrsf21, Itgb6, Sp1, Aqp7, Suclg2, Chd9, Vwa7, Mkx, Cntnap5c, Olfr1151, Kcne1, Stxbp6, Sv2c, Zfp560, Slc35d1, Tox2, Dlgap1, Olfr599
mt-tRF3a-ProTGG	Cplx2, Emp2, Fam124a, Olfr357, Gcm1, Vmn1r66, Il21r, Pde3b, 1500009C09Rik, Fat3, Atp1a2, Olfr1262, Olfr160, Pigr, Olfr1122, Ms4a12, Slco3a1, Pdzd9, Runx2, Polr3b

## Discussion

In our previous study, vitamin D deficiency was observed in children with greater adiposity during the first 5 years of life^5^. However, the mehcanism has not been well-defined. In recent years, Plenty of studies revealed that vitamin D deficiency is closely related to obesity or adipose tissue through intricate mechanisms. the mechanisms have shown that vitamin D in adipose tissue exerted influences on lipolysis and adipogenesis^14^, adipocyte differentiation^15^, energy metabolism^15^, adipose tissue development and function^16^, oxidative stress^17^, inflammation^18^, secretion of adipocytokines^19^, lipid metabolism^20^, proliferation and apoptosis and thermogenesis^21^, the catabolic and anabolic activity of adipocytes^22^, adipose tissue DNA hypomethylation^23^, and the expression of microRNAs^24–26^ by regulating different signaling pathways and metabolic processes. However, The expression of tsRNA in WAT of VD deficiency young male mice with or without obesity has not been reported.

In recent years, the research on tsRNA and disease disorders has gradually become a hot topic, mainly focusing on tumor diseases such as gastric cancer, lung cancer and hepatocellular carcinoma, neurodegenerative disorders and so on^27,28^. However, there are no reports on the characteristics and changing patterns of tsRNA expression in WAT of VD deficiency young male mice with or without obesity, beside, the role and mechanism are not clear. In this study, tsRNAs for the first time were identified in subcutaneous adipose tissues of VD deficiency young male mice with or without obesity.

The high-throughput sequencing technology was used to analyze the differential expression of tsRNA and found that tRF5-20-HisGTG-3 was highly expressed and mt-tRF3a-ProTGG was low expressed not only in WAT of mice fed with high-fat VD deficiency diet compared with high-fat VD sufficiency diet, but aslo in WAT of mice fed with high-fat VD deficiency diet compared with low-fat VD sufficiency diet. Quantitative PCR further verified that no matter combined with obesity or not, tRF5-20-HisGTG-3 were significantly up-regulated and mt-tRF3a-ProTGG were significantly down-regulated in VD deficiency mouses. It was assumed that tRF5-20-HisGTG-3 and mt-tRF3a-ProTGG may participate in the regulation of VD deficiency. Changwon Yang et al^29^. verified quercetin regulated the expression of tiRNAHisGTG, and transfection of a tiRNAHisGTG mimic further enhanced the apoptotic effect of quercetin in colorectal cancer cells. Bayazit M B^30^et al. identified tiRNA-5HisGTG was enriched in mitochondrial fractions of β cells, tiRNA-5HisGTG inhibition in these islets reduced b cell proliferation and insulin secretion, and mitochondrial respiration was also perturbed. Bourgery M^31^et al. provided evidence on the levels of 5′tiRNA His-GTG-5′ and i-tRF His-GTG were increased during fracture healing. Su Z et al. found that angiogenin overexpression selectively cleaved tRNAHis^32^. Taxis T M et al^33^. suggests that tRF5HisGTG could potentially be used as biomarkers to establish exposure of cattle to Bovine leukemia virus. In addition, the study of mt-tRF3a-ProTGG has not been reported.

While, the high-throughput sequencing technology found that tRF5-22-CysGCA-27 was highly expressed, and mt-5’tiRNA-33-AlaTGC, mt-5’tiRNA-33-SerTGA and mt-5’tiRNA-32-SerTGA were low expressed not only in WAT of mice fed with high-fat VD deficiency diet compared with high-fat VD sufficiency diet, but aslo in WAT of mice fed with high-fat VD deficiency diet compared with low-fat VD sufficiency diet.

Quantitative PCR further verified that tRF5-22-CysGCA-27 were significantly up-regulated and mt-5’tiRNA-33-AlaTGC, mt-5’tiRNA-33-SerTGA and mt-5’tiRNA-32-SerTGA were significantly down-regulated only in WAT of mice fed with high-fat VD deficiency diet compared with low-fat VD sufficiency diet. This means, the relative expressions of tRF5-22-CysGCA-27, mt-5’tiRNA-33-AlaTGC, mt-5’tiRNA-33-SerTGA and mt-5’tiRNA-32-SerTGA were greatly influenced by both circulating VD concerntrtion and degree of fatness, but the molecular mechanism need to be further investigated. The study demonstrated that the tRNA-Cys-GCA suppress tumor progression of gliomas^34^. Elevation in plasma 5’AlaTGC precede seizures in human epilepsy^35^. Ayan G B et al^36^. postulate that tRNA-Ser(CGA) elimination increases the translational demand for tRNA-Ser(UGA), a pressure relieved by increasing serTGA copy number.

The high-throughput sequencing technology found that tRF3a-GlyGCC-1 were highly expressed only in WAT of mice fed with high-fat VD deficiency diet compared with low-fat VD sufficiency diet. Quantitative PCR further verified that tRF3a-GlyGCC-1 were significantly up-regulated only in WAT of mice fed with high-fat VD deficiency diet compared with low-fat VD sufficiency diet. Several studies showed that 5’-tRF-GlyGCC promoted breast tumorigenesis and metastasis^37^, and was correlated with an aggressive phenotype of ovarian tumor^38^, and was a novel biomarker for colorectal cancer diagnosis^39^. our study found that tRF3a-GlyGCC-1 were greatly influenced by both circulating VD concerntrtion and degree of fatness. This provides a theoretical basis for further study of its molecular mechanism of action, but it still needs further experimental verification.

Here, we first explored the tsRNA profiles in WAT of VD deficiency mouses with or without obesity. On the basis of comparisons between different groups, the complicated regulatory mechanism was preliminarily revealed. Further more, the results was relatively persuasive due to the previous population-based study including large sample data. At last, high-throughput sequencing technology possesses the advantages of high sensitivity and high accuracy, which made the results more informative. This research could provide a new strategy for the intervention treatment of VD deficiency associated obesity. However, several limitations exsist in this study. Firstly, the PCR verification of tsRNAs was insufficient in this study. Secondly, we explored the tsRNA profiles in samples, but the further mechanism remained undefined, and the absence of functional verification made the results less convincing.

## Conclusions

The tsRNAs were differentially expressed in VD deficiency with obesity, especially tRF5-20-HisGTG-3, tRF5-22-CysGCA-27, tRF3a-GlyGCC-1, mt-5’tiRNA-33-AlaTGC, mt-5’tiRNA-33-SerTGA, mt-5’tiRNA-32-SerTGA and mt-tRF3a-ProTGG. These tsRNAs seemed to be associated with FoxO signaling pathway, GnRH secretion, 2 − oxocarboxylic acid metabolism, autophagy, glucagon and insulin signaling pathway, pathways of neurodegeneration − multiple diseases, metabolic process and biological regulation.

## Supplementary Information

Below is the link to the electronic supplementary material.

Supplementary Material 1

## Data Availability

The data that support the findings of this study are available from the corresponding author but restrictions apply to the availability of these data, which were used under license from Institutional Animal Care and Use Committee at Nanjing Medical University for the current study, and so are not publicly available. Data are, however, available from the authors upon reasonable request and with permission from Institutional Animal Care and Use Committee at Nanjing Medical University.

## Declarations

### Statement

Our study is reported in accordance with ARRIVE guidelines.
