# Supplementary material for: Expression profile of tsRNAs in white adipose tissue of vitamin D deficiency young male mice with or without obesity
Source: Sci Rep. 2024 Nov 11;14:27486. doi: 10.1038/s41598-024-77910-9 (PMC11551137; doi:10.1038/s41598-024-77910-9)
Supplement: Supplementary file 1 — Supplementary Material 1 [file 41598_2024_77910_MOESM1_ESM.doc]

**Table S1. Main components of feed in each group**

| Group | The proportion of energy supplied by fat | VD_3_ content |
| --- | --- | --- |
| HFVDD group | 60% | 0～25 IU/3606 kcal |
| HFVDS group | 60% | 1000 IU/3606 kcal |
| ConVDS group | 10% | 1000 IU/3606 kcal |
| ConVDD group | 10% | 0～25 IU/3606 kcal |

**Table S2. The body weight and serum VD_3_ levels of the mice at week 8**

| HFVDD | | HFVDS | | ConVDD | | ConVDS | |
| --- | --- | --- | --- | --- | --- | --- | --- |
| Weight | 25-(OH)D | Weight | 25-(OH)D | Weight | 25-(OH)D | Weight | 25-(OH)D |
| 34.2 | <7.500 | 25.6 | 52.1 | 26.1 | 12.7 | 25.6 | 42.9 |
| 34.4 | <7.500 | 25.4 | 48.2 | 27.4 | 16.0 | 25.2 | 51.5 |
| 34.3 | <7.500 | 28.3 | 47.9 | 28.1 | 10.8 | 25.7 | 51.2 |

**Table S3. RNA Quantification and Quality Assurance by NanoDrop ND-1000**

| Sample ID | A260/A280  ratio | A260/A230  ratio | Concentration  (ng/ul) | Volume  (ul) | Quality  (ng) |
| --- | --- | --- | --- | --- | --- |
| HFVDD-1 | 1.87 | 2.28 | 350.45 | 15 | 5256.75 |
| HFVDD-2 | 1.99 | 1.85 | 750.72 | 40 | 30028.80 |
| HFVDD-3 | 1.95 | 1.98 | 579.43 | 20 | 11588.60 |
| HFVDS-1 | 1.96 | 1.99 | 638.75 | 40 | 25550.00 |
| HFVDS-2 | 1.99 | 2.32 | 1108.14 | 30 | 33244.20 |
| HFVDS-3 | 1.97 | 1.99 | 587.87 | 15 | 8818.05 |
| ConVDD-1 | 1.98 | 1.98 | 1112.08 | 30 | 33362.40 |
| ConVDD-2 | 1.97 | 2.08 | 706.39 | 40 | 28255.60 |
| ConVDD-3 | 1.98 | 2.40 | 1230.46 | 20 | 24609.20 |
| ConVDS-1 | 1.96 | 2.41 | 881.64 | 20 | 17632.80 |
| ConVDS-2 | 1.93 | 2.41 | 495.90 | 20 | 9918.00 |
| ConVDS-3 | 1.94 | 2.11 | 700.08 | 20 | 14001.60 |

**Table S4. The differentially expressed tsRNAs identified among the four groups**

| Group | Num up. Sig | Num down. Sig | Num diff. Sig |
| --- | --- | --- | --- |
| HFVDD/HFVDS | 37 | 18 | 55 |
| HFVDD/ConVDD | 34 | 50 | 84 |
| HFVDD/ConVDS | 55 | 101 | 156 |
| HFVDS/ConVDD | 84 | 131 | 215 |
| HFVDS/ConVDS | 76 | 54 | 130 |
| ConVDD/ConVDS | 18 | 25 | 43 |

Num up: the number of up-regulated tsRNAs (FC ≥ 1.5); Num down: the number of down-regulated tsRNAs (FC ≤ 0.67); Num diff: the total number of differentically expressed tsRNAs; Num up. Sig: the number of significantly up-regulated tsRNAs (FC ≥ 1.5, p < 0.05); Num down. Sig: the number of significantly down-regulated tsRNAs (FC ≤ 0.67, p < 0.05); Num diff. Sig: the total number of significantly differentically expressed tsRNAs.


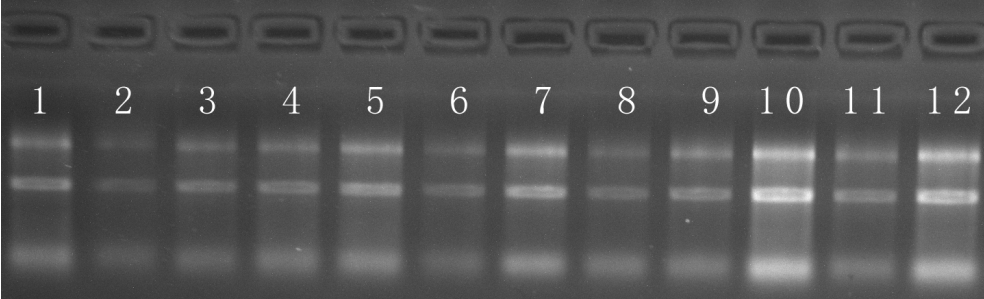


Figure S1. RNA Integrity and gDNA contamination test by Denaturing Agarose Gel Electrophoresis
